# Supplementary material for: Processing bodies promote lysosomal quality control and cell survival during recovery from lysosomal damage
Source: Res Sq. 2025 Sep 9:rs.3.rs-7474186. Preprint. [Version 1] doi: 10.21203/rs.3.rs-7474186/v1 (PMC12440071; doi:10.21203/rs.3.rs-7474186/v1)
Supplement: 1 [file NIHPPRS7474186V1-supplement-1.pdf]

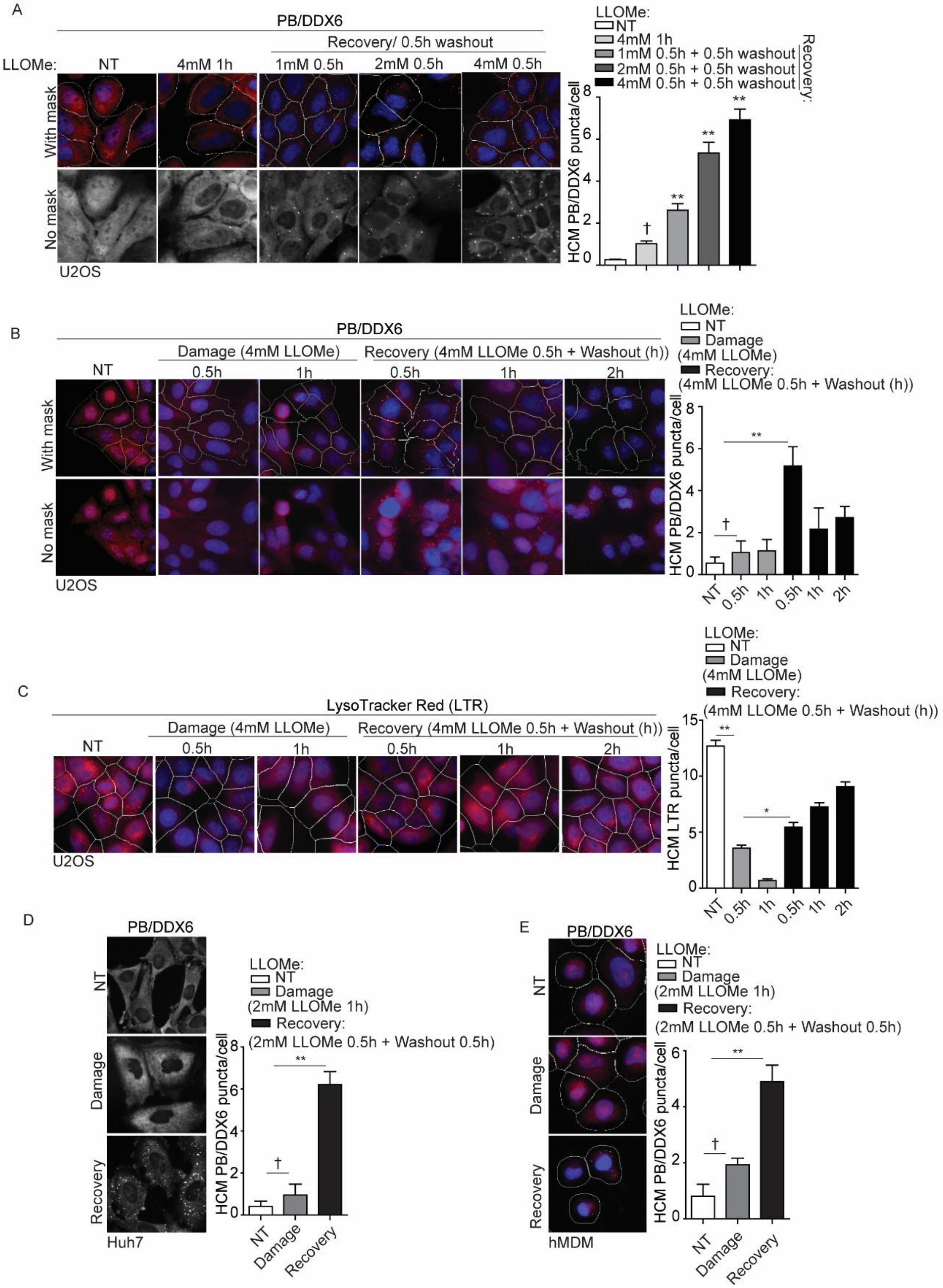

Figure S1. Processing body formation is associated with recovery from lysosomal damage.

(A) Quantification of the processing body (PB) marker DDX6 by high-content microscopy (HCM) in U2OS cells treated with 4 mM LLOMe for 1 h to induce damage, or with a dose-dependent LLOMe treatment for 30 min, followed by a 30 min recovery after washout. White masks, algorithm-defined cell boundaries; red masks, computer-identified DDX6 puncta. No mask image is shown in grayscale. **(B)** Quantification of the PB marker DDX6 by HCM in U2OS cells treated with 4 mM LLOMe for 30 min or 1 h to induce damage, or with 4 mM LLOMe for 30 min, followed by a time-dependent recovery. White masks, algorithm-defined cell boundaries; red masks, computer-identified DDX6 puncta. **(C)** Status of acidified organelles in U2OS cells assessed by LysoTracker (LTR) staining and HCM during treatment as described in (B). White masks, algorithm-defined cell boundaries; red masks, computer-identified LTR puncta. **(D)** Quantification of the PB marker DDX6 by HCM in Huh7 cells treated with 2 mM LLOMe for 1 h to induce damage, or for 30 min followed by a 30 min recovery after washout. The image is shown in grayscale. **(E)** Quantification of the PB marker DDX6 by HCM in human peripheral blood monocyte-derived macrophages (hMDM) treated with 2 mM LLOMe for 1 h to induce damage, or for 30 min followed by a 30 min recovery after washout. White masks, algorithm-defined cell boundaries; red masks, computer-identified DDX6 puncta. NT, untreated cells. Data, means ± SEM (n = 3); HCM: n ≥ 3 (each experiment: 500 valid cells per well, ≥5 wells/sample). † $p \geq 0.05$ (not significant), *$p < 0.05$, **$p < 0.01$, ANOVA. See also Figure 1.

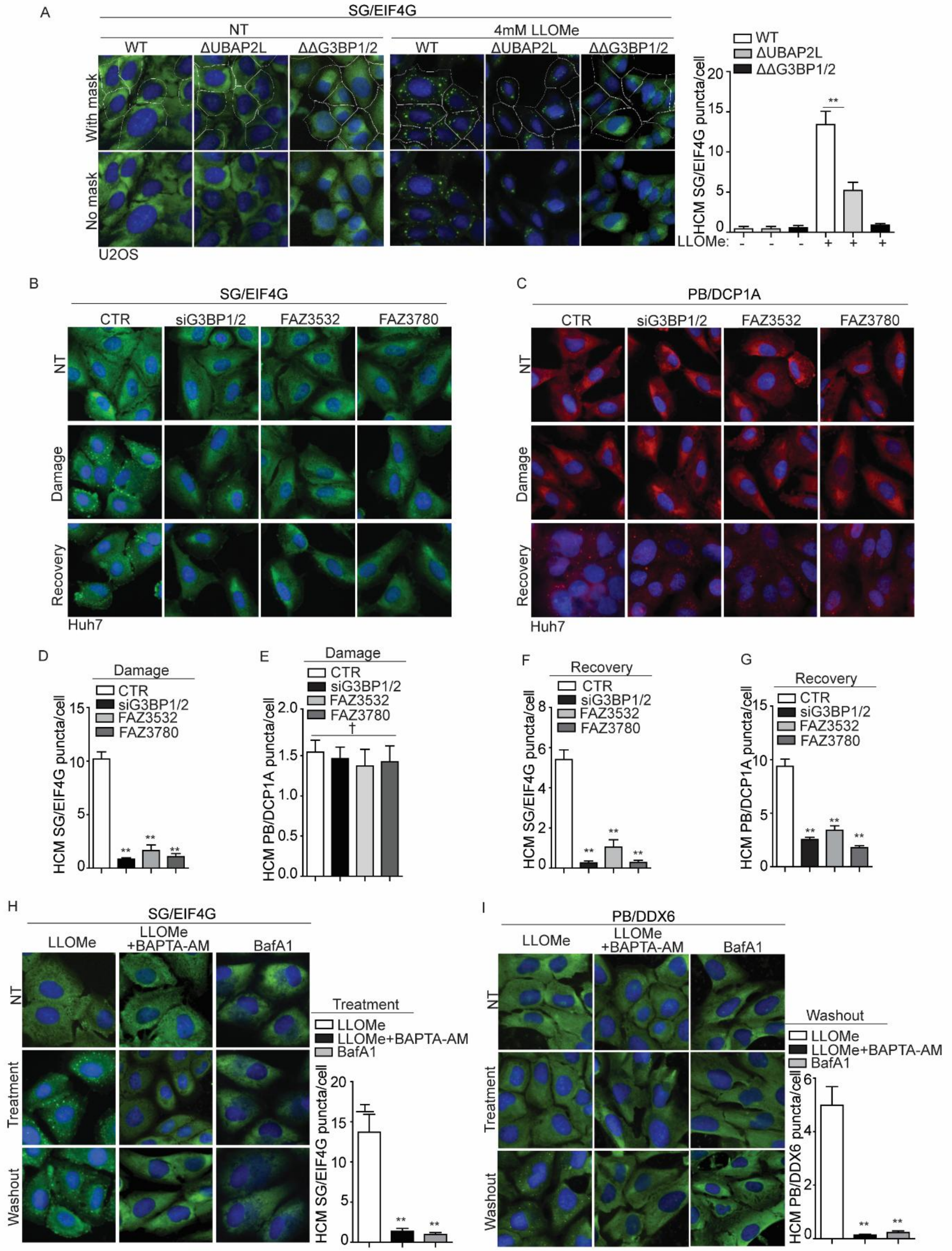

Figure S2. Stress granules facilitate processing body formation during recovery from lysosomal damage. (A) Quantification of the SG marker EIF4G by HCM in U2OS wildtype (WT), UBAP2L knockout (ΔUBAP2L), and G3BP1&2 double knockout (ΔΔG3BP1/2) cells treated with

4 mM LLOMe for 1 h. White masks, algorithm-defined cell boundaries; green masks, computer-identified EIF4G puncta. **(B)** Quantification of the stress granule (SG) marker EIF4G by HCM in Huh7 cells transfected with either scrambled siRNA as control (CTR) or siRNA targeting G3BP1 and G3BP2 for knockdown (siG3BP1/2), or treated with SG inhibitors (20 μM FAZ3532/FAZ3780). Cells were treated with 4 mM LLOMe for 1 h to induce damage, or for 30 min followed by a 30 min recovery after washout. **(C)** Quantification of the PB marker DCP1A by HCM in Huh7 cells transfected with either scrambled siRNA as control (CTR) or siRNA targeting G3BP1 and G3BP2 for knockdown (siG3BP1/2) or treated with SG inhibitors. Cells were treated with 4 mM LLOMe for 1 h to induce damage, or for 30 min followed by a 30 min recovery after washout. **(D-G)** Quantification of data shown in (B) and (C). Quantification of the SG marker EIF4G **(H)** and the PB marker DDX6 **(I)** by HCM in U2OS cells. Cells were treated with 4 mM LLOMe with or without 15 μM BAPTA-AM, or with 100 nM Bafilomycin A1(BafA1) for 1 h, followed by a 30 min recovery after washout. NT, untreated cells. Data, means ± SEM (n = 3); HCM: n ≥ 3 (each experiment: 500 valid cells per well, ≥5 wells/sample). † $p \geq 0.05$ (not significant), **$p < 0.01$, ANOVA. See also Figure 2.

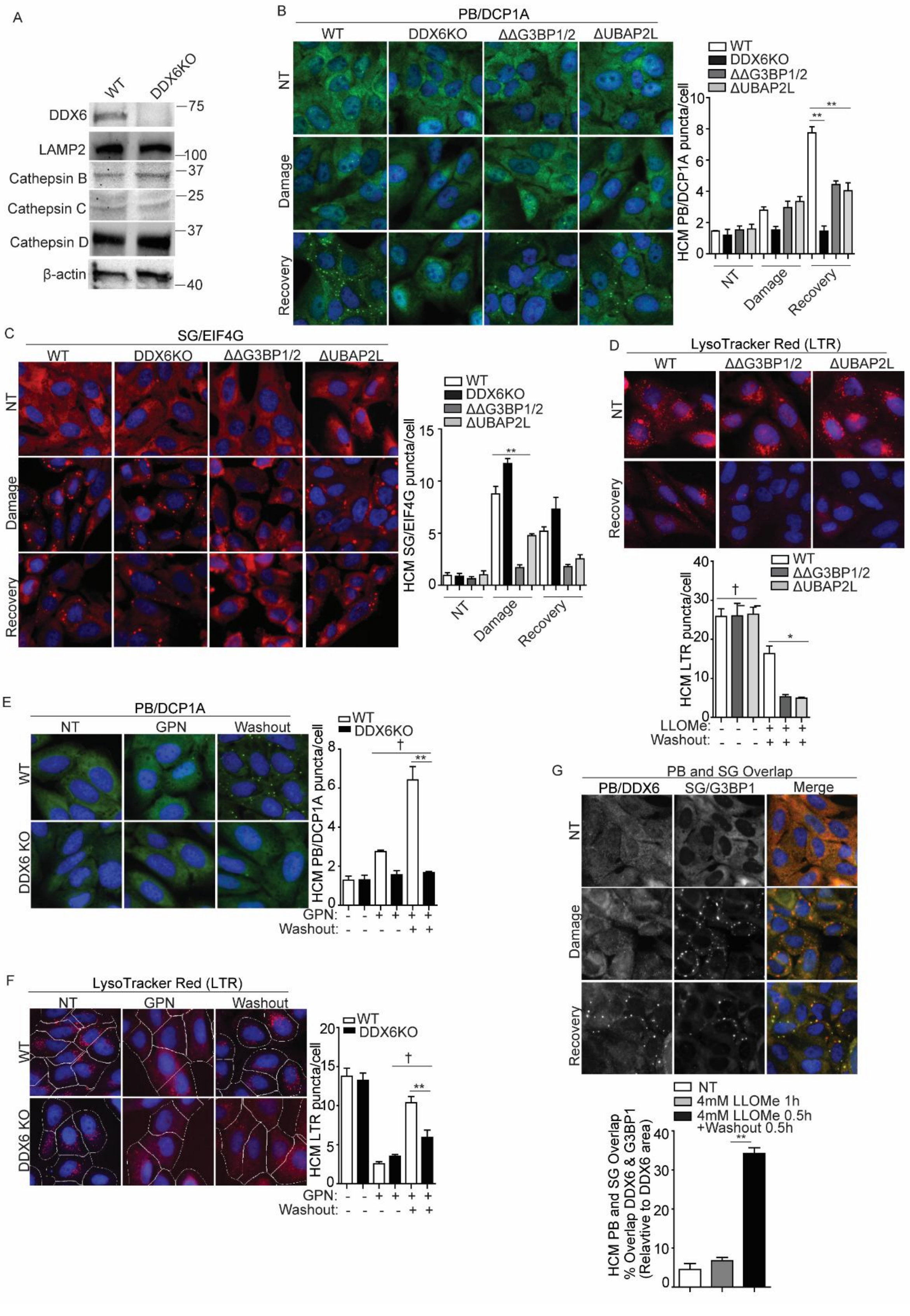

A
WT
DDX6KO
DDX6
LAMP2
Cathepsin B
Cathepsin C
Cathepsin D
β-actin
75
100
37
25
37
40
B
PB/DCP1A
WT
DDX6KO
ΔΔG3BP1/2
ΔUBAP2L
NT
Damage
Recovery
HCM PB/DCP1A puncta/cell
C
SG/EIF4G
HCM SG/EIF4G puncta/cell
D
LysoTracker Red (LTR)
HCM LTR puncta/cell
LLOMe:
Washout:
E
PB/DCP1A
NT
GPN
Washout
DDX6 KO
GPN:
Washout:
F
LysoTracker Red (LTR)
G
PB and SG Overlap
PB/DDX6
SG/G3BP1
Merge
4mM LLOMe 1h
4mM LLOMe 0.5h
+Washout 0.5h
HCM PB and SG Overlap
% Overlap DDX6 & G3BP1
(Relavtive to DDX6 area)

Figure S3. Processing body and stress granule formation during lysosomal recovery is important for maintaining lysosomal quality. (A) Immunoblot analysis of the indicated proteins in U2OS wildtype (WT) and DDX6 knockout (DDX6KO) cells. **(B)** Quantification of the PB marker DCP1A (Alexa Fluor 488) by HCM in U2OS wildtype (WT), DDX6 knockout (DDX6KO), G3BP1&2 double knockout (ΔΔG3BP1/2) and UBAP2L knockout (ΔUBAP2L) cells treated with 2 mM LLOMe for 1 h to induce damage or treated for 30 min followed by a 30 min recovery after washout. **(C)** Quantification of the SG marker EIF4G (Alexa Fluor 568) by HCM in U2OS wildtype (WT), DDX6 knockout (DDX6KO), G3BP1&2 double knockout (ΔΔG3BP1/2) and UBAP2L knockout (ΔUBAP2L) cells treated with 4 mM LLOMe for 1 h to induce damage or treated for 30 min followed by a 30 min recovery after washout. **(D)** Status of acidified organelles in U2OS wildtype (WT), G3BP1&2 double knockout (ΔΔG3BP1/2) and UBAP2L knockout (ΔUBAP2L) cells assessed by LysoTracker (LTR) staining and HCM during recovery (2 mM LLOMe for 30 min followed by a 30 min recovery after washout). **(E)** Quantification of the PB marker DCP1A by HCM in U2OS wildtype (WT) and DDX6 knockout (DDX6KO) cells treated with 200 μM GPN for 1 h to induce damage, or for 30 min followed by a 30 min recovery after washout. **(F)** Status of acidified organelles in U2OS wildtype (WT) and DDX6 knockout (DDX6KO) cells assessed by LysoTracker (LTR) staining and HCM during lysosomal damage (200 μM GPN for 1 h) or recovery (200 μM GPN for 30 min followed by a 30 min recovery after washout). White masks, algorithm-defined cell boundaries; red masks, computer-identified LTR puncta. **(G)** HCM quantification of overlaps between the PB marker DDX6 (Alexa Fluor 488) and the SG marker G3BP1 (Alexa Fluor 568) in U2OS cells treated with 4 mM LLOMe for 1 h to induce damage, or for 30 min followed by a 30 min recovery after washout. NT, untreated cells. Data, means ± SEM (n = 3); HCM: n ≥ 3 (each experiment: 500 valid cells per well, ≥5 wells/sample). † p ≥ 0.05 (not significant), *p < 0.05, **p < 0.01, ANOVA. See also Figure 3.

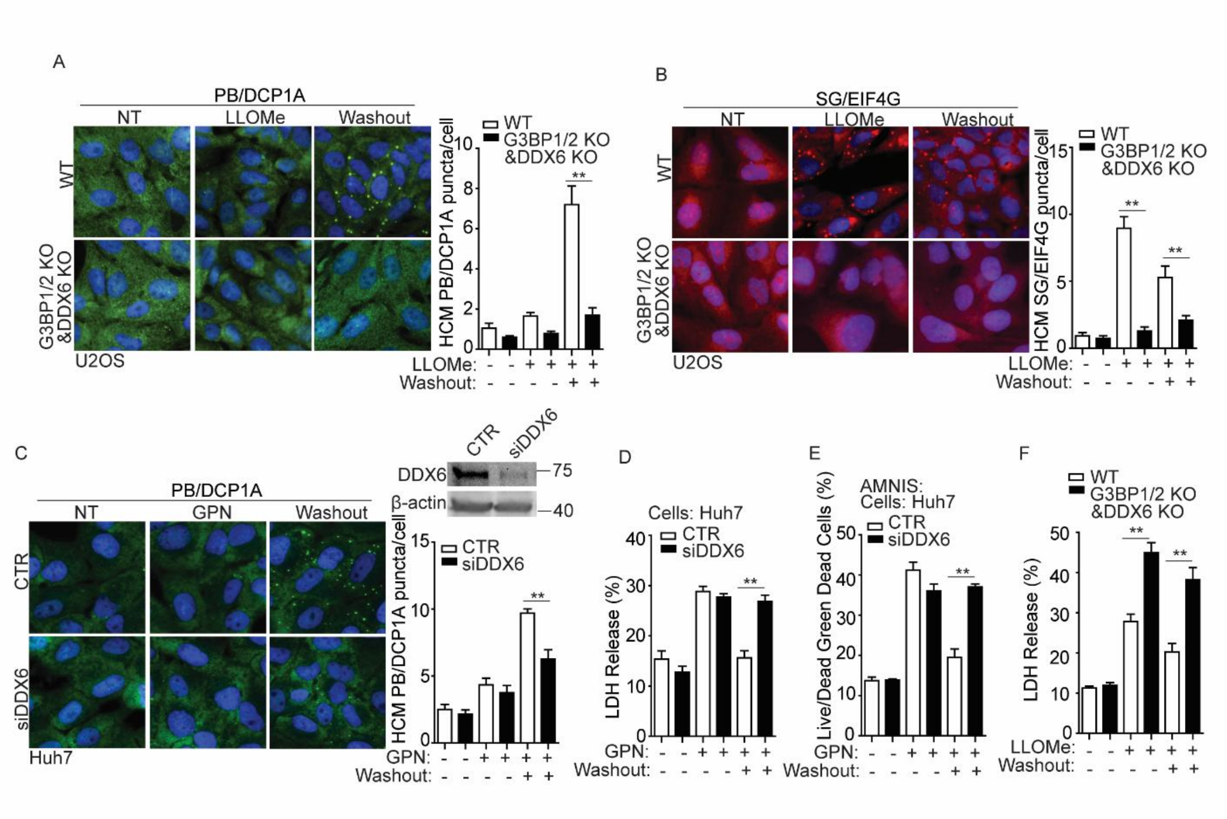

Figure S4. Processing body formation during lysosomal recovery is important for cell survival. (**A)** Quantification of the PB marker DCP1A (Alexa Fluor 488) by HCM in U2OS wildtype (WT) and G3BP1/2 and DDX6 double knockout (G3BP1/2KO&DDX6KO) cells. Cells were treated with 4 mM LLOMe for 1 h to induce damage, or for 30 min followed by a 30 min recovery after washout. (**B)** Quantification of the SG marker EIF4G (Alexa Fluor 568) by HCM in U2OS wildtype (WT) and G3BP1/2 and DDX6 double knockout (G3BP1/2KO&DDX6KO) cells. Cells were treated with 4 mM LLOMe for 1 h to induce damage, or for 30 min followed by a 30 min recovery after washout. **(C)** Quantification of the PB marker DCP1A (Alexa Fluor 488) by HCM in Huh7 cells transfected with either scrambled siRNA as control (CTR) or siRNA targeting DDX6 for knockdown (siDDX6). Cells were treated with 200 µM GPN for 1 h to induce damage, or for 30 min followed by a 30 min recovery after washout. (**D**) Cell death analysis of supernatants from Huh7 cells transfected with either scrambled siRNA as control (CTR) or siRNA targeting DDX6 for knockdown (siDDX6) by an LDH release assay. Cells were treated as described in (C). (**E**) Quantification of cell death using the AMNIS system and Live/Dead™ stain kit in Huh7 cells transfected with either scrambled siRNA as control (CTR) or siRNA targeting DDX6 for knockdown (siDDX6). Cells were treated as described in (C) and then stained using Live/Dead™ stain kit (ThermoFisher). (**F**) Cell death analysis of supernatants from U2OS wildtype (WT) and G3BP1/2 and DDX6 double knockout (G3BP1/2KO&DDX6KO) cells by a LDH release assay. Cells were treated as described in (A). NT, untreated cells. Data, means ± SEM (n = 3); HCM: n ≥ 3 (each experiment: 500 valid cells per well, ≥5 wells/sample). **p < 0.01, ANOVA. See also Figure 4.
